# Distinction of High- and Low-Frequency Repetitive Transcranial Magnetic Stimulation on the Functional Reorganization of the Motor Network in Stroke Patients

**DOI:** 10.1155/2021/8873221

**Published:** 2021-01-20

**Authors:** Zhiwei Guo, Yu Jin, Xi Bai, Binghu Jiang, Lin He, Morgan A. McClure, Qiwen Mu

**Affiliations:** ^1^Department of Radiology, Institute of Rehabilitation and Imaging of Brain Function, The Second Clinical Medical College of North Sichuan Medical College, Nanchong Central Hospital, Nanchong, Sichuan, China 637000; ^2^Department of Radiology, Langzhong People's Hospital, Langzhong, China 637400; ^3^Department of Radiology, Peking University Third Hospital, Beijing, China 100191

## Abstract

**Objective:**

To investigate the functional reorganization of the motor network after repetitive transcranial magnetic stimulation (rTMS) in stroke patients with motor dysfunction and the distinction between high-frequency rTMS (HF-rTMS) and low-frequency rTMS (LF-rTMS).

**Methods:**

Thirty-three subcortical stroke patients were enrolled and assigned to the HF-rTMS group, LF-rTMS group, and sham group. Each patient of rTMS groups received either 10.0 Hz rTMS over the ipsilesional primary motor cortex (M1) or 1.0 Hz rTMS over the contralesional M1 for 10 consecutive days. A resting-state functional magnetic resonance imaging (fMRI) scan and neurological examinations were performed at baseline and after rTMS. The motor network and functional connectivities intramotor network with the core brain regions including the bilateral M1, premotor area (PMA), and supplementary motor area (SMA) were calculated. Comparisons of functional connectivities and Pearson correlation analysis between functional connectivity changes and behavioral improvement were calculated.

**Results:**

Significant motor improvement was found after rTMS in all groups which was larger in two rTMS groups than in the sham group. The functional connectivities of the motor network were significantly increased in bilateral M1, SMA, and contralesional PMA after real rTMS. These changes were only detected in the regions of the ipsilesional hemisphere in the HF-rTMS group and in the regions of the contralesional hemisphere in the LF-rTMS group. Significantly changed functional connectivities of the intramotor network were found between the ipsilesional M1 and SMA and contralesional PMA, between contralesional M1 and contralesional SMA, between contralesional SMA and ipsilesional SMA and contralesional PMA in the HF-rTMS group in which the changed connectivity between ipsilesional M1 and contralesional PMA was obviously correlated with the motor improvement. In addition, the functional connectivity of the intramotor network between ipsilesional M1 and contralesional PMA was significantly higher in the HF-rTMS group than in the LF-rTMS group.

**Conclusion:**

Both HF-rTMS and LF-rTMS have a positive effect on motor recovery in patients with subcortical stroke and could promote the reorganization of the motor network. HF-rTMS may contribute more to the functional connectivity reorganization of the ipsilesional motor network and realize greater benefit to the motor recovery.

## 1. Introduction

Interhemispheric imbalance and reduced interactions of neural activity and functional connectivity have been reported in both animal and human studies after stroke with motor dysfunction [1–4]. In addition, as the level of impairment increased, the network balance was more disrupted [5]. Therefore, the balance of the motor network between the two brain hemispheres is crucial for functional motor recovery of stroke patients [6]. Noninvasive brain stimulation, e.g., repetitive transcranial magnetic stimulation (rTMS), has been recognized as an effective strategy to facilitate motor recovery by enhancing/suppressing neural excitability of ipsilesional/contralesional hemispheres to restore interhemispheric balance [7–9]. Finally, these lead to cerebral plasticity and reorganization of the motor network of the damaged hemisphere.

Numerous functional neuroimaging studies have confirmed that recovery of motor function after stroke is commonly attributed to cortical reorganization of both ipsilesional sensorimotor areas and contralesional motor areas [10–13]. This reorganization is adaptive and is gradually shifted during the process of regaining motor function in the affected limbs. Additionally, reorganization of the ipsilesional hemisphere is traditionally believed to be most important for successful recovery [14]. Findings from a study of low-frequency rTMS (LF-rTMS) over the contralesional primary motor cortex (M1) suggested that one single session of rTMS could transiently remodel the architecture of the disturbed motor network, reflected as reduced transcallosal influences and a restitution of ipsilesional functional connectivity, in particular, the effective connectivity between M1 and supplementary motor area (SMA) [15]. Another stroke study with long-term high-frequency rTMS (HF-rTMS) treatment observed increased interhemispheric functional connectivity between ipsilesional M1 and contralesional motor areas [16]. Dual-mode stimulation combined with transcranial direct current stimulation (tDCS) also detected noticeably increased interhemispheric connectivity in subacute stroke patients [17]. However, in these studies, the difference between HF-rTMS and LF-rTMS on the influence of functional reorganization of the motor network was still not clear. The relationship between motor network reorganization and motor improvement has not been clarified. Maybe the restoration of some part of the motor network showed greater contribution to the recovery of motor function than others.

Therefore, to further clarify the reorganization of interhemispheric and intrahemispheric functional connectivity of the motor network and the relationship with motor recovery of rTMS, this study was aimed at investigating the connectivity changes between brain regions of the motor network after HF-rTMS or LF-rTMS. The comparison of the motor network changes after HF-rTMS and LF-rTMS was also conducted to ascertain their different modulation mechanisms on the motor network. We hypothesized that significantly increased functional connectivities and their correlation with motor improvement would be observed in some motor areas after HF-rTMS or LF-rTMS. The influence on the motor network may be distinct between them.

## 2. Materials and Methods

### 2.1. Participants

Thirty-three right-handed stroke patients (mean age: 64.48, range 53-78 years) with motor deficits after a first-onset subcortical ischemic stroke in the territory of the left middle cerebral artery were enrolled from the Department of Neurology at the Second Clinical Medical College of North Sichuan Medical College (Nanchong, China) according to the following inclusion criteria: (1) right handedness, (2) ischemic lesion at the unilateral subcortical area confirmed by diffusion-weighted imaging (DWI), (3) showing unilateral motor dysfunction, (4) no history of neurological/psychiatric diseases, and (5) no contraindications of rTMS and MRI measurement. Exclusion criteria were as follows: (1) hemorrhagic stroke, (2) any other brain disorder or abnormalities, (3) history of drug dependency or psychiatric disorders, (4) severe white matter hyperintensity, (5) substantial head movement during the fMRI data acquisition according to the preprocessing result, and (6) contraindication to MRI and/or TMS.

According to the Helsinki Declaration, this study was approved by the Ethics Committee of the Second Clinical Medical College of North Sichuan Medical College. This study was registered in the Chinese Clinical Trial Registry (ChiCTR-IOR-16008629) and reported following the guidelines of the Consolidated Standards of Reporting Trials (CONSORT) group. All participants gave informed consent before the experiment.

### 2.2. Study Design

All stroke patients were enrolled at the acute stage with a subcortical lesion location encompassing the left internal capsule, basal ganglia, or corona radiate. These patients were assigned to the HF-rTMS group (11 subjects, five males and six females, mean age 65.09 ± 5.84, range 58-75 years), LF-rTMS group (12 subjects, five males and seven females, mean age 63.58 ± 7.95, range 53-78 years), and sham group (10 subjects, five males and five females, mean age 64.90 ± 6.23, range 58-75 years). Each patient received rTMS daily for 10 consecutive days. An MRI scan and several comprehensive neurological examinations including the National Institutes of Health Stroke Scale (NIHSS), Fugl-Meyer Assessment (FMA), and Barthel Index (BI) were performed prior to the experiment and immediately after 10 days of rTMS. Based on these scales, the stroke severity, motor impairment, and daily living ability were evaluated.

### 2.3. Intervention

After stroke, the equilibrium of cortical excitability between the two hemispheres is disrupted. This has shown decreased excitability of the ipsilesional hemisphere and increased excitability of the contralesional hemisphere [18]. Based on the interhemispheric competition model, previous studies have reported that the inhibitory rTMS on the contralesional hemisphere could increase excitability of the ipsilesional motor cortex by reducing excessive interhemispheric inhibition from the contralesional motor cortex [19, 20], whereas excitatory rTMS over the affected hemisphere directly increases the excitability of the ipsilesional motor cortex [21, 22]. Therefore, the strategy of HF-rTMS over the ipsilesional motor cortex and LF-rTMS over the contralesional motor cortex was selected in our study.

rTMS was performed by using a Magpro R30 stimulator (MagVenture, Lucernemarken, Denmark) equipped with a 70.0 mm butterfly-shape coil and a handle posterior and oriented sagittally. The scalp site that could elicit response in the first dorsal interosseous muscle of the affected/unaffected hand was selected as the optimal location of the center of the rTMS coil for HF-rTMS/LF-rTMS intervention. If nonresponsive activity could be detected stimulating the ipsilesional M1 for the patients in the HF-rTMS group, symmetric location homologous to the contralesional M1 would be defined as the stimulation site. A resting motor threshold (RMT) was established and was defined as the lowest rTMS intensity that could elicit a motor-evoked potential of at least an amplitude of 50 𝜇V in at least half of 10 consecutive stimuli over the M1 [23]. Stimulation was applied at 90% RMT at 1.0 Hz frequency (900 pulses) over contralesional M1 in the LF-rTMS group (30 trains, 30 pulses/train, intertrain interval = one second, and a total of 900 pulses) and at 90% RMT at 10.0 Hz frequency (30 trains, 50 pulses/train, intertrain interval = 25 seconds, and a total of 1,500 pulses) over ipsilesional M1 in the HF-rTMS group. The sham group received rTMS with the same parameters as the LF-rTMS group over the contralesional M1 but without real stimulation to ensure that no current flow was induced in the brain. All rTMS sessions were performed in the same room. All stroke patients received the same physiotherapy and medical therapies which consisted of standard antiplatelet, statin, anticoagulation, and antihypertensive drugs during the period spent in hospital.

### 2.4. MRI Acquisition

The resting-state fMRI data were acquired on a GE Signa HDxt 1.5 Tesla scanner (General Electric Medical System, Milwaukee, WI, USA) with an eight-channel head coil. To reduce head movements and scanner noises, the head of each patient was snugly fixed by a foam pad prior to the examination. After instructing the patients to keep awake, relaxed with eyes closed, and to remain motionless as much as possible, functional magnetic resonance imaging (fMRI) data were acquired by using an echo-planar imaging (EPI) sequence: TR/TE = 2, 000/40 ms, field of view = 240.0 × 240.0 mm^2^, flip angle = 90°, matrix = 64 × 64, voxel sizes = 3.75 × 3.75 × 5.0 mm^3^, 32 axial slices, and no gaps. Each scan obtained 140 volumes continuously. A 3D high-resolution structural image acquisition was also conducted: 124 slices, TR/TE = 9.1/2.9 ms, field of view = 240.0 × 240.0 mm^2^, flip angle = 20°, matrix = 256 × 256, and voxel sizes = 0.94 × 0.94 × 1.2 mm^3^.

### 2.5. Preprocessing of the fMRI Data

Image preprocessing was performed by using the SPM 12 (http://www.fil.ion.ucl.ac.uk/spm) software package. Prior to the preprocessing procedure, the first five volumes of the fMRI datasets of each patient were discarded to eliminate the magnetization equilibrium effects and allow the participants to adapt to the circumstances. Subsequently, spatial processing including time delay correction between slices, head motion realignment, spatial normalization to the standard brain space of the Montreal Neurological Institute (MNI) (resampled to a voxel size of 3.0 × 3.0 × 3.0 mm), and spatial smoothing with 8.0 mm isotropic kernel was conducted.

### 2.6. Independent Component Analysis

Only the fMRI data of both rTMS groups was used to analyze the difference between HF-rTMS and LF-rTMS on the modulation of the motor network. With the preprocessed fMRI data, the GIFT software (http://icatb.sourceforge.net/) was used to conduct the group spatial independent component analysis (ICA) with the following stages: (1) two-stage data reduction of principal component analysis (PCA), (2) application of the ICA algorithm, and (3) back reconstruction using a dual-regression method to back reconstruct the individual independent components (ICs). To determine the number of ICs, dimension estimation on all patients of both rTMS groups was performed by using the minimum description length (MDL) criterion. Subsequently, the infomax algorithm was used in IC estimation. Then, following the reconstruction step, the individual specific IC maps were converted to a *Z* score. At last, the IC of the motor network was selected to be of interest for further analyses. *Z* maps of each group were then gathered for a random effects analysis using the one-sample *t*-test in SPM 12. Subsequently, to investigate the functional connectivity changes of the motor network after rTMS, the paired *t*-test analysis was used to compare the *Z* maps of the motor network of both groups between pre- and post-rTMS. Moreover, the same comparison of the *Z* maps between pre- and post-rTMS was conducted for each group, respectively, and also to understand the distinction of functional connectivity changes between the HF-rTMS and LF-rTMS groups.

### 2.7. Functional Connectivity Analysis of the Intramotor Network

Motor recovery of stroke has been demonstrated to be associated with the reorganization of the functional motor network [24]. Consistent dynamically increased regional centralities of the ipsilesional M1 within the motor network was also observed with the process of motor recovery [25]. Therefore, in this study, the core regions of the cortical motor network of bilateral hemispheres including M1, SMA, and premotor area (PMA) were mainly focused on in order to investigate the modulation of rTMS on the functional connectivities among these regions of the intramotor network. The peak coordinates of these core regions were identified and selected from the comparison results of the motor network obtained from ICA analysis between pre- and post-rTMS of both groups. Finally, a spherical region of interest (ROI) (radius = 5.0 mm) was defined and centered at each peak coordinate within the corresponding brain region.

Subsequently, the signal extraction, preprocessing, and functional connectivity analysis of the motor network were all completed in the Resting-State Hemodynamic Response Function Retrieval and Deconvolution (rsHRF) plugin (https://github.com/compneuro-da/rsHRF) in SPM [26]. By using this software package, the blood oxygenation level-dependent (BOLD) fMRI signal was deconvolved to minimize the variability of HRF [27]. The time series of all the voxels in each ROI was extracted from the preprocessed fMRI dataset and averaged as the representative time signal of the ROI. To minimize the effect of global drift, the time signal of each ROI was scaled by dividing each time point's value by the mean value of the whole brain image at that time point. After this, the scaled waveform of each signal was filtered by using a bandpass filter (0.01-0.08 Hz) to reduce the effect of low-frequency drift and high-frequency artifacts related to head motion and physiological noise including respiration and cardiac cycle. The head motion parameters, white matter signals, and cerebrospinal fluid signals were then used as covariates of multiple linear regression. Subsequently, the Pearson correlation coefficients were calculated between the time signals of all ROIs and normalized to *z*-scores by using Fisher's *r* to *z* transformation. Statistically significant (*p* < 0.05) correlation coefficient was considered a valid connectivity and used to describe the edge of the motor network. For each patient, two motor networks were obtained pre- and post-rTMS. A paired *t*-test was employed to observe the significantly changed connectivities between regions after rTMS for the HF-rTMS group and LF-rTMS group separately.

### 2.8. Correlation Analysis

To further verify the consistent performance between the functional connectivity of the motor network and motor function, we computed the Pearson correlation coefficients between the values of functional connectivity changes and motor assessment score changes as well in each group. The statistical analysis was conducted by using a threshold of *p* < 0.05.

### 2.9. Statistical Analysis

Statistics for demographics and cognitive test scores were calculated with appropriate chi-squared (*χ*^2^), ANCOVA, or Student's *t*-tests. Statistical parametric and nonparametric tests were used depending on the type of scale and nature of the variable distribution. ANCOVA with age and gender as covariates was performed to determine the main effect of rTMS, followed by post hoc two-sample *t*-tests for multiple comparisons. Paired *t*-tests were conducted to assess the changes of cognitive function postintervention within each group. The significance was set at *p* < 0.05.

## 3. Results

### 3.1. Behavioral Information

The demographic characteristics and neurological examinations of HF-rTMS, LF-rTMS, and sham groups are summarized in Table 1. The mean and standard deviation (SD) of age, the time since stroke (days), and the FMA, BI, and NIHSS of patients of pre- and post-rTMS are all provided in the table. There are no significant differences among the three groups in age, gender, time since stroke (days), or clinical performances at baseline. Compared to baseline, both the motor function and daily living ability postintervention were all significantly improved according to the results of the two-factor ANCOVA which revealed significant main effects of “time” for the FMA, BI, and NIHSS (*p* < 0.001). The significant interaction between “group” and “time” was also found for the FMA (*F* = 13.023, *p* < 0.001) and BI (*F* = 6.021, *p* = 0.006) scores. Post hoc *t*-tests revealed that NIHSS scores were significantly lower in both rTMS groups compared to the sham group (HF-rTMS vs. sham, *p* = 0.028; LF-rTMS vs. sham, *p* = 0.020). The paired *t*-test revealed significantly improved FMA, BI, and NIHSS scores in the three groups after rTMS treatment relative to pre-rTMS (*p* < 0.05). All the score changes of FMA, BI, and NIHSS scores after rTMS were bigger in the HF-rTMS group relative to LF-rTMS and sham groups. During the rTMS sessions, no discomfort was reported from any patients in three groups.

### 3.2. Changes of Functional Connectivity of the Motor Network

**Figure 1:**
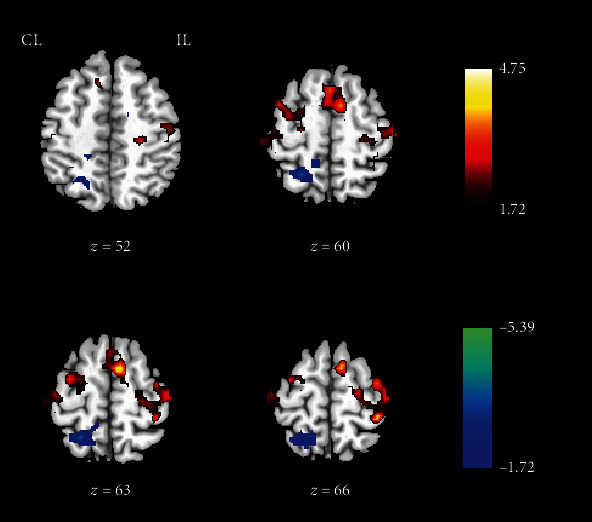
Functional connectivity changes of the motor network after rTMS treatment. CL: contralesional side; IL: ipsilesional side. The warm color indicates the increased functional connectivity, and the cold color indicates the decreased functional connectivity after rTMS.

**Figure 2:**
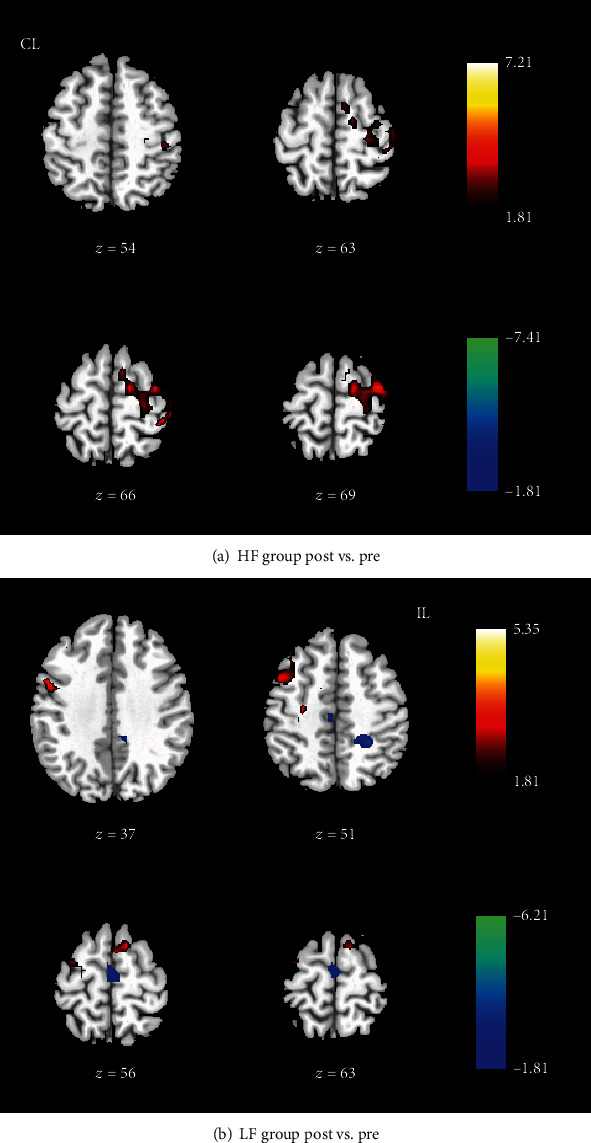
Functional connectivity changes of the motor network after HF-rTMS (a) and LF-rTMS (b) separately. CL: contralesional side; IL: ipsilesional side. The warm color indicates the higher functional connectivity, and the cold color indicates the lower functional connectivity in the HF-rTMS group.

After the group ICA analysis, the spatial independent component image of the motor network was extracted for each patient. These image data of both HF-rTMS and LF-rTMS groups were used to investigate the influence of rTMS therapy on the functional connectivity of the motor network. Compared to pre-rTMS, the significantly increased functional connectivity was observed in bilateral M1, SMA, and contralesional PMA after rTMS (*p* < 0.05, AlphaSim correction, and cluster size > 197) (Figure 1 and Table 2). In addition, to further clarify the distinction of HF-rTMS and LF-rTMS on the modulation of functional connectivity of the motor network, respectively, the comparison between pre- and post-rTMS in the HF group and LF-rTMS group was performed separately. Significantly increased functional connectivity was observed in the ipsilesional M1, SMA, and PMA after HF-rTMS (*p* < 0.05, AlphaSim correction, and cluster size > 219) (Figure 2(a)). In contrast, the enhanced functional connectivities were observed in the contralesional M1 and bilateral SMA in the LF-rTMS group after rTMS (*p* < 0.05, AlphaSim correction, and cluster size > 213) (Figure 2(b)). Furthermore, decreased functional connectivity was detected in the bilateral SMA as well.

### 3.3. Changes of Functional Connectivities of the Intramotor Network

**Figure 3:**
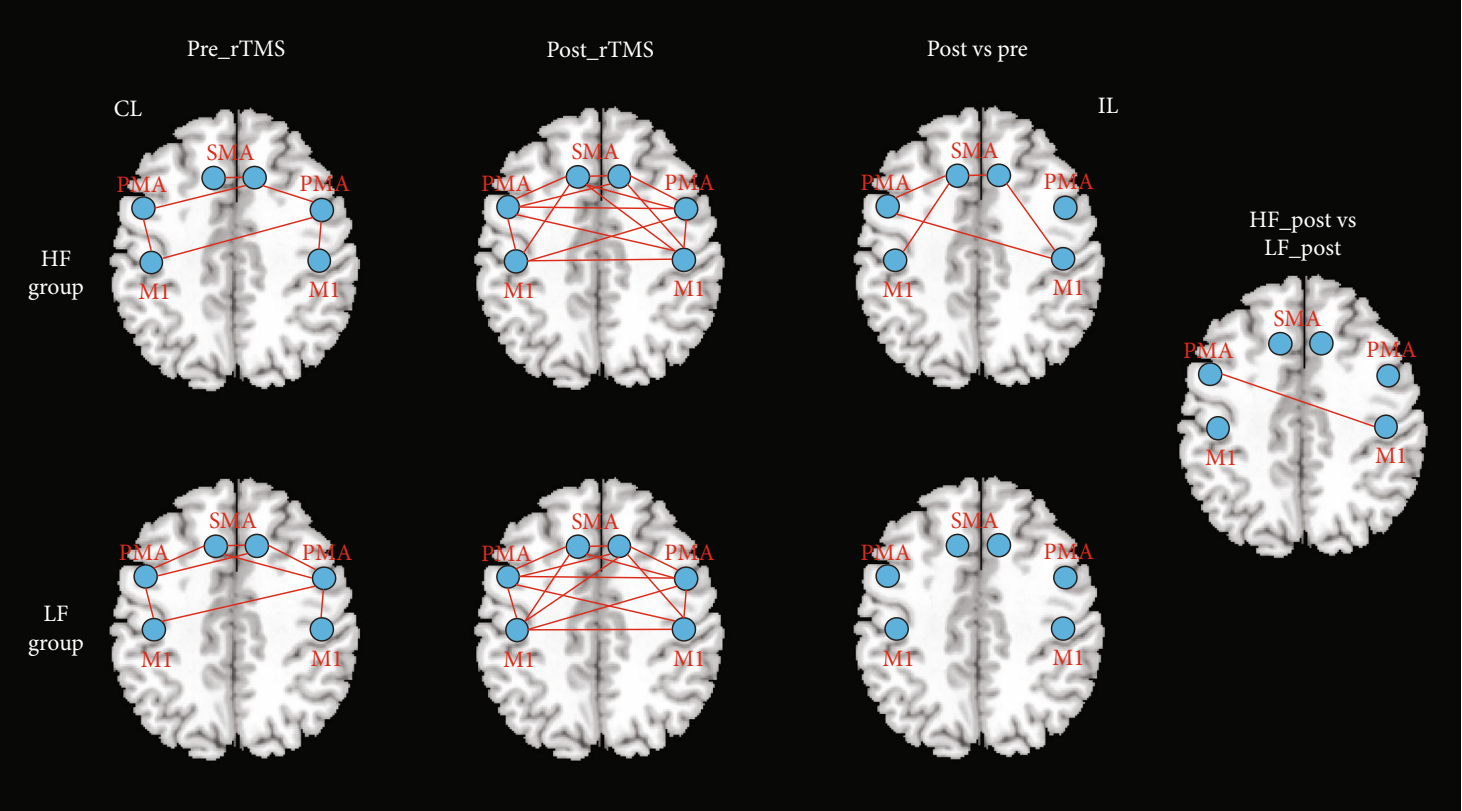
Significant functional connectivity intramotor network and changes after rTMS. HF: high frequency; LF: low frequency; CL: contralesional side; IL: ipsilesional side; M1: primary motor cortex; SMA: supplementary motor area; PMA: premotor area.

To validate the modulation of rTMS on the network pathway between brain regions of the motor network, the functional connectivity intramotor network was calculated with the selected peak coordinates in Table 2. The symmetric location homologous to the contralesional PMA (-33, -7, and 61) and SMA (9, 2, and 61) was selected for the two regions which did not show significant changes after rTMS. The comparisons of functional connectivity of the intramotor network pre- and post-rTMS within each group and between HF-rTMS and LF-rTMS groups after rTMS were also conducted.  Figure 3 demonstrates statistically significant functional connectivity and changes of the motor network pre- and post-rTMS in the HF-rTMS group and LF-rTMS group and between two groups. The disconnectivity induced by stroke at baseline was basically recovered after rTMS, especially among the ipsilesional motor-related brain regions and between regions of the ipsilesional and contralesional hemisphere. Although most of the connectivity did not reach a statistically significant level, these findings revealed the reconnection within the motor network of the affected hemisphere and with the unaffected hemisphere after rTMS.

The significantly increased functional connectivities were detected between the ipsilesional M1, ipsilesional SMA, and contralesional PMA, between contralesional M1 and contralesional SMA, and between contralesional SMA, ipsilesional SMA, and contralesional PMA in the HF-rTMS group. No significant functional connectivity changes were observed in the LF-rTMS group. Significantly higher functional connectivity was found between ipsilesional M1 and contralesional PMA in HF-rTMS relative to the LF-rTMS group as well. These findings suggest the modulation of rTMS on functional interactions among the motor brain regions within the affected hemisphere and interaction of bilateral hemispheres following treatment.

### 3.4. Relationship between Functional Connectivity and Motor Performance

**Figure 4:**
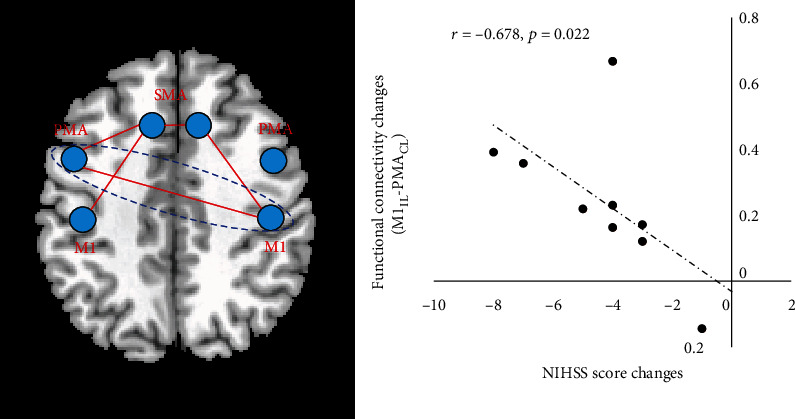
Pearson correlation between the changes of functional connectivity (between ipsilesional PMA and contralesional M1) and NIHSS score changes in the HF-rTMS group. M1: primary motor cortex; PMA: premotor area; SMA: supplementary motor area; IL: ipsilesional; CL: contralesional.

To verify the relationship between the significantly changed functional connectivity and motor recovery alteration reflected by neurological examination, a Pearson correlation coefficient was calculated in both HF-rTMS and LF-rTMS groups. For the functional connectivity intramotor network, the increased functional connectivity between ipsilesional M1 and contralesional PMA (*r* = −0.678, *p* = 0.022) (Figure 4) was significantly negatively correlated with the NIHSS improvement in the HF-rTMS group. No significant correlation result was detected in the LF-rTMS group and other functional connectivities of the motor network. This result may indicate the reconnection between the brain regions which may contribute to the restoration of motor function after HF-rTMS.

## 4. Discussion

In this current study, both ICA and seed-based analyses were used to investigate the functional reorganization of the motor network of stroke patients with motor deficit after rTMS. The distinction between HF-rTMS and LF-rTMS on the modulation of the motor network was further discussed. We found that HF-rTMS prominently increased the functional connectivity of the motor network in the ipsilesional hemisphere, whereas LF-rTMS mainly focused on the contralesional hemisphere. Moreover, the interaction between ipsilesional M1 and contralesional PMA and between bilateral SMA may contribute more during the motor recovery with HF-rTMS therapy. Our findings suggest that the distinct functional restoration and reorganization within the motor network of HF-rTMS and LF-rTMS both may underlie the motor recovery.

In our study, significantly improved motor function was detected in both HF-rTMS and LF-rTMS groups relative to baseline and sham groups. Furthermore, greater changes of FMA, BI, and NIHSS were all found in the HF-rTMS group than in the patients in the LF-rTMS group. The positive effect of rTMS on the motor recovery and activities of daily living of stroke patients with motor dysfunction has been reported in several meta-analyses [7, 28, 29]. In accordance with our results, one of the meta-analyses also found that HF-rTMS is more effective than LF-rTMS, but not significant [28]. However, the opposite result was reported in another meta-analysis [7]. Therefore, future investigation with more studies is necessary to validate the result.

Consistent with the results of neurological examinations, significantly increased functional connectivity of the motor network was observed in both groups as well. Furthermore, the motor-related brain regions showing network changes were located in the ipsilesional hemisphere after HF-rTMS and in the contralesional hemisphere after LF-rTMS. These results could be explained with the distinct mechanisms of different modes of rTMS which suggested that HF-rTMS over the ipsilesional hemisphere could increase the cortical excitability of the damaged cortex; low-frequency rTMS over the contralesional hemisphere could potentially decrease abnormally increased inhibition to the lesioned M1 and promote the recovery of the damaged cortex [30]. Several comprehensive studies on motor recovery in early stroke patients showed that both HF-rTMS and LF-rTMS could increase motor-evoked fMRI activation of the ipsilesional motor area which were also positively significantly correlated with motor function at postintervention in M1 [31–33]. The increased fMRI activation in ipsilesional M1 was observed in patients with good motor outcome as well [31]. Therefore, both the excited rTMS over the ipsilesional M1 and the inhibitory rTMS over the contralesional hemisphere have shown promise in enhancing stroke patients' recovery [14].

Except for different motor network changes, more significant functional connectivities intramotor network was found in the HF-rTMS group between the ipsilesional motor cortex and contralesional motor areas. The increased functional connectivity between ipsilesional M1 and contralesional PMA was also observed significantly related to the motor improvement. Additionally, this connectivity was also found higher in the HF-rTMS group than in the LF-rTMS group. Several previous studies have proved the crucial role of contralesional PMA, in particular, the dorsal PMA, in motor function and motor recovery. After stroke, fMRI investigations showed more activation in the contralesional PMA during the movement of the affected limb and were prominent in patients with poor motor recovery [34–36]. Such activity changes may imply the associated motor recovery. Inhibitory low-frequency rTMS over contralesional PMA also could slow the affected finger movement, in particular in more impaired patients, suggesting the functional recruitment of contralesional PMA in motor recovery [36]. This results also demonstrated its adaptive compensation for an injured motor cortex after stroke. Further studies on behavior, neuroimaging, and neuropsychological validate that motor impairment and recovery after stroke could be explained with the specificity of PMA to the process of action selection [37–39]. Moreover, a concurrent TMS-fMRI study further found the physiological influence of contralesional PMA on ipsilesional M1 [40]. Furthermore, stronger promotional influence between them was associated with greater clinical and neuropsychological impairment during hand grip in stroke patients. Dual-site TMS studies also found that TMS-induced activation changes in contralesional PMA have a causal impact on ipsilesional M1 at short latencies [41, 42], so a likely alternative route by which contralesional PMA could exert control over ipsilesional finger movement is via interhemispheric connections with contralateral M1 [43]. Therefore, these evidences suggest that contralesional PMA may be positioned to mediate functional recovery of motor function after stroke. The finding of significantly increased functional connectivity between ipsilesional M1 and contralesional PMA after rTMS may be explained by these above-mentioned theories and prove its contribution to motor recovery during high-frequency rTMS therapy.

Significant functional connectivity between ipsilesional and contralesional M1 was also observed after rTMS in both HF-rTMS and LF-rTMS groups, which was impaired after stroke. A previous study reported that increased functional connectivity between bilateral M1 was significantly correlated with the improvement in the upper limb section of FMA which was detected after the motor imagery training combined with conventional rehabilitation therapy [44]. Another study with acupuncture treatment also observed increased functional connectivity between bilateral M1 [45]. In addition, prior to treatment, several studies found significantly decreased interhemispheric functional connectivity between ipsilesional M1 and contralesional M1 after stroke [4, 45–47]. One study suggested that the transcallosal connections between bilateral M1 was also associated with motor recovery [48]. Therefore, our finding may indicate the efficacy and modulatory effect of high- and low-frequency rTMS on the motor network.

In considering the whole brain, stroke induces interhemispheric changes and not just the neural activity and functional connectivity in the affected and unaffected hemisphere [49]. Therefore, according to the model of interhemispheric interaction, motor recovery after stroke may be linked to rebalancing of asymmetric interhemispheric excitability and connectivity. This theory also confirmed the rationale of neuromodulation techniques to suppress unaffected motor cortex excitability and facilitate affected motor cortex excitability [50]. Noninvasive treatments including rTMS and transcranial direct current stimulation (tDCS) were both mainly performed to restore abnormal interhemispheric balance by facilitating ipsilesional M1 excitability or by inhibiting contralesional M1 excitability [17, 22, 51, 52]. They observed slightly but not significantly increased intrahemispheric connectivity of the ipsilesional M1 after stimulation with both rTMS and tDCS [17, 53]. This is in accordance with our results between the ipsilesional M1 and PMA. The functional role of SMA for motor recovery has been proven for a long time. The functional connectivity increase between the ipsilesional M1 and contralesional SMA demonstrated the efficacy of rTMS. Moreover, significant changes in neurochemicals were detected in the affected M1 as well when stimulating the unaffected M1. They believed that interhemispheric connectivity is also particularly important in functional recovery after stroke. In our study, more interhemispheric functional connectivity changes were observed which may indicate that functional compensation from the contralesional hemisphere may play a more important role during motor recovery. rTMS may realize its effect by modulating the functional connectivities between ipsilesional and contralesional motor-related brain areas. Direct intervention of HF-rTMS over the affected M1 may contribute more to the motor recovery which could explain the more increased functional connectivity of the motor network.

Some limitations exist in our study. First, a relatively small sample size was used in our study which may influence the results. We only included 11 subjects for the HF-rTMS group, 12 subjects for the LF-rTMS group, and 10 subjects for the sham group. It is difficult to ensure the cohorts of patients, but, in this study, there was no significant difference among the three groups in demographic characteristics, neurological examinations, and functional connectivity at baseline. Studies with more stroke patients are needed to verify our results. Second, only the core regions of the motor network were selected to characterize the functional reorganization. Subcortical brain regions also could be considered to fully understand the network changes after rTMS. Third, after completing the arranged sessions, the durability and influence on the motor network of HF-rTMS and LF-rTMS interventions were not made with the postintervention measurements.

Therefore, further studies with large sample sizes and long-term follow-up assessments are needed to interpret and verify the results more accurately.

## 5. Conclusions

Our study demonstrates that both HF-rTMS and LF-rTMS interventions could promote the motor rehabilitation in patients with stroke. Strikingly, HF-rTMS over the ipsilesional M1 may be more beneficial to the reorganization of the motor network and remodeling of motor cortical plasticity which realize greater contribution to the motor recovery.

## Figures and Tables

**Table 1:** Demographic, clinical, and motor test variables of stroke patients.

Variables		HF_group (*n* = 11)	LF_group (*n* = 12)	Sham_group (*n* = 10)	*F*/*χ*^2^	*p*
Age		65.09 ± 5.84	63.58 ± 7.95	64.9 ± 6.23	0.168	0.846
Gender (F/M)		6/5	7/5	5/5	0.153	0.926
Time since stroke (days)		6.00 ± 2.37	5.42 ± 1.93	5.1 ± 1.79	0.528	0.595
FMA	Pre	38.45 ± 22.64	37.83 ± 15.06	36.70 ± 15.37	13.023	0.000
	Post	54.64 ± 19.82^a,b^	52.67 ± 19.98^a,b^	40.6 ± 16.33^a,b^		
BI	Pre	43.64 ± 25.31	45.42 ± 20.05	43.00 ± 15.49	6.021	0.006
	Post	61.82 ± 21.71^a,b^	59.58 ± 21.24^a,b^	47.50 ± 13.59^a,b^		
NIHSS	Pre	7.09 ± 2.77	5.75 ± 2.73	7.40 ± 1.96	2.852	0.073
	Post	3.27 ± 1.74^a^	3.17 ± 2.66^a^	5.40 ± 1.71^a^		

HF: high frequency; LF: low frequency; FMA: Fugl-Meyer Assessment; BI: Barthel Index; NIHSS: National Institutes of Health Stroke Scale; M: male; F: female. ^a^The significant differences between pre- and post-rTMS with a paired *t*-test (*p* < 0.05). ^b^The significant differences between groups from baseline to postintervention with repeated measures ANOVA (*p* < 0.05).

**Table 2:** Brain regions showing significantly changed functional connectivities in the motor network after rTMS in both rTMS groups.

Region	Side	*T* value	Cluster size (voxels)	MNI coordinate		
				*x*	*y*	*z*
M1	IL	4.11	298	-39	-37	64
M1	CL	2.55	123	45	-19	61
SMA	BL	4.27	187	-9	2	61
PMA	CL	3.34	151	33	-7	61

MNI: Montreal Neurological Institute; M1: primary motor cortex; SMA: supplementary motor cortex; PMA: premotor area; IL: ipsilesional side; CL: contralesional side; BL: bilateral side.

## Data Availability

The behavioral data used to support the findings of this study and the statistical analysis results are included within the supplementary information file. The data of fMRI used to support the findings of this study have not been made available because of the large number of original image files.

## Supplementary Materials

Supplementary Materials Original data of the basic information and behavioral scores of enrolled patients.
