# Supplementary material for: Distinction of High- and Low-Frequency Repetitive Transcranial Magnetic Stimulation on the Functional Reorganization of the Motor Network in Stroke Patients
Source: Neural Plast. 2021 Jan 20;2021:8873221. doi: 10.1155/2021/8873221 (PMC7840259; doi:10.1155/2021/8873221)
Supplement: Supplementary Materials — Original data of the basic information and behavioral scores of enrolled patients. [file 8873221.f1.docx]

**Supplementary Material – Original data**

**Supp. Table 1. Information of the patient in low-frequency rTMS group**

| **NO.** | **Gender** | **Age** | **Foci** | **Duration** | **Rest Motor Threshold** | **FM** | | **BI** | | **NIHSS** | |
| --- | --- | --- | --- | --- | --- | --- | --- | --- | --- | --- | --- |
|  |  |  |  |  |  | **Pre** | **Post** | **Pre** | **Post** | **Pre** | **Post** |
| 1 | M | 54 | L_BG | 2 | 48% | 55 | 74 | 55 | 70 | 5 | 2 |
| 2 | F | 56 | L_CR | 5 | 53% | 29 | 48 | 80 | 95 | 2 | 1 |
| 3 | F | 61 | L_BG | 4 | 50% | 39 | 48 | 50 | 60 | 7 | 5 |
| 4 | M | 68 | L_BG | 6 | 43% | 56 | 81 | 45 | 60 | 4 | 2 |
| 5 | M | 78 | L_BG | 6 | 43% | 58 | 69 | 85 | 95 | 3 | 1 |
| 6 | F | 67 | L_IC, BG, CR | 5 | 45% | 34 | 50 | 30 | 55 | 7 | 3 |
| 7 | M | 74 | L_BG | 9 | 51% | 22 | 28 | 25 | 30 | 10 | 7 |
| 8 | F | 61 | L_BG | 6 | 43% | 46 | 64 | 30 | 60 | 6 | 1 |
| 9 | F | 58 | L_IC, BG, CR | 6 | 51% | 18 | 27 | 35 | 50 | 9 | 8 |
| 10 | F | 53 | L_IC | 5 | 48% | 44 | 66 | 50 | 65 | 5 | 2 |
| 11 | M | 70 | L_BG | 8 | 50% | 13 | 18 | 25 | 30 | 2 | 0 |
| 12 | F | 63 | L_BG, CR | 3 | 49% | 40 | 59 | 35 | 45 | 9 | 6 |

**Supp. Table 2. Information of the patient in high-frequency rTMS group**

| **NO.** | **Age** | **Gender** | **Foci** | **Duration** | **Rest Motor Threshold** | **FM** | | **BI** | | **NIHSS** | |
| --- | --- | --- | --- | --- | --- | --- | --- | --- | --- | --- | --- |
|  |  |  |  |  |  | **Pre** | **Post** | **Pre** | **Post** | **Pre** | **Post** |
| 1 | 70 | F | L_IC, BG | 5 | 57% | 23 | 42 | 20 | 40 | 9 | 5 |
| 2 | 63 | F | L_IC, BG | 5 | 55% | 77 | 86 | 60 | 80 | 6 | 2 |
| 3 | 62 | F | L_BG, CR | 8 | 43% | 27 | 39 | 50 | 50 | 5 | 4 |
| 4 | 59 | M | L_IC, BG | 5 | 50% | 14 | 40 | 20 | 50 | 9 | 2 |
| 5 | 73 | M | L_BG | 9 | 46% | 59 | 69 | 45 | 70 | 4 | 2 |
| 6 | 75 | F | L_BG | 3 | 55% | 32 | 56 | 35 | 50 | 10 | 6 |
| 7 | 64 | M | L_IC, BG | 6 | 50% | 36 | 58 | 25 | 70 | 11 | 3 |
| 8 | 64 | F | L_BG | 9 | 55% | 22 | 25 | 25 | 30 | 7 | 2 |
| 9 | 59 | M | L_BG, IC | 9 | 40% | 56 | 70 | 90 | 95 | 2 | 1 |
| 10 | 69 | F | CR | 3 | 45% | 67 | 80 | 85 | 95 | 6 | 3 |
| 11 | 58 | M | L_IC, BG | 4 | 54% | 10 | 36 | 25 | 50 | 9 | 6 |

**Supp. Table 3. Information of the patient in sham rTMS group**

| **NO.** | **Age** | **Gender** | **Duration** | **Foci** | **FMA** | | **BI** | | **NIHSS** | |
| --- | --- | --- | --- | --- | --- | --- | --- | --- | --- | --- |
|  |  |  |  |  | **Pre** | **Post** | **Pre** | **Post** | **Pre** | **Post** |
| 1 | 73 | F | 4 | IC, BG | 45 | 51 | 55 | 60 | 7 | 5 |
| 2 | 59 | M | 9 | IC, BG | 64 | 70 | 75 | 75 | 4 | 3 |
| 3 | 62 | F | 7 | IC, BG | 32 | 36 | 40 | 45 | 8 | 8 |
| 4 | 72 | F | 6 | IC,BG,CR | 21 | 24 | 30 | 35 | 6 | 5 |
| 5 | 63 | F | 4 | BG | 45 | 48 | 45 | 50 | 9 | 5 |
| 6 | 59 | M | 4 | IC, BG | 38 | 41 | 50 | 55 | 10 | 4 |
| 7 | 75 | M | 3 | IC, BG | 20 | 24 | 35 | 40 | 9 | 7 |
| 8 | 64 | F | 5 | IC,BG | 54 | 58 | 50 | 50 | 7 | 4 |
| 9 | 64 | M | 5 | IC,BG | 30 | 35 | 25 | 35 | 5 | 5 |
| 10 | 58 | M | 4 | IC,BG,CR | 18 | 19 | 25 | 30 | 9 | 8 |
